# Bowel Obstruction as the Initial Presentation of Urothelial Carcinoma

**DOI:** 10.7759/cureus.64056

**Published:** 2024-07-08

**Authors:** Francisco Girão de Caires, Mafalda Nunes, Priscila Flores, António Girão de Caires, Isabel Dionísio

**Affiliations:** 1 General Surgery, Centro Hospitalar do Oeste, Caldas da Rainha, PRT; 2 Intensive Care Unit, Centro Hospitalar Médio Tejo, Abrantes, PRT; 3 General Surgery, Hospital Central do Funchal, Funchal, PRT

**Keywords:** bowel obstruction, oncologic surgery, en bloc surgery, emergency abdominal surgery, high grade urothelial carcinoma

## Abstract

Bowel obstructions are one of the main causes of hospital admissions for acute abdominal pain. In addition, bladder cancer is one of the most common cancers in the world. This said, bowel obstruction and bladder cancer are very frequent diseases but the same cannot be said about the association between these two pathologies.

We report a unique case of an 80-year-old patient admitted to the emergency room with a bowel obstruction caused by a urothelial carcinoma with adrenal metastasis.

The patient underwent an urgent laparotomy, and intraoperative inspection of the peritoneal cavity confirmed a large tumorous mass suspected of gastrointestinal etiology. The mass infiltrated the ileum and sigmoid colon and was apparently in contact with the bladder wall. An en-bloc resection of the lesion was performed. An R0 excison was not possible and fragments of the lesion were excised from the bladder wall for separate analysis.

Histopathological examination of the resected specimen described a high-grade, undifferentiated urothelial carcinoma that originated in the bladder and invaded the ileum and sigmoid colon.

The presence of an invasive urothelial carcinoma presenting with bowel obstruction represents an unexpected diagnosis and, although rare, the surgeon must be aware of this possibility. This case should serve as a reminder that a broad differential diagnosis should be considered when investigating an abdominal tumor.

## Introduction

Bowel obstructions are responsible for approximately 15% of hospital admissions for acute abdominal pain [1], but only approximately 20% of cases need surgical care [1]. In 90% of cases, small bowel obstruction is caused by adhesions, hernias, and neoplasms [1]. An adhesive small bowel obstruction represents 55-75% of these cases [2] while hernias and small bowel tumors account for the remainder [3].

The main cause of large bowel obstruction is cancer in about 60% of cases [4], with volvulus and diverticular disease being responsible for the other 30% [5]. The remaining 10-15% of causes include carcinomatosis, endometriosis, inflammatory bowel disease, stenosis, and other organ malignancies, as described in this article [6].

Bladder cancer (BC) is one of the most common cancers in the world. In 2020, it was responsible for over 210,000 deaths, and approximately 570,000 new cases have arisen worldwide [7]. That said, bowel obstruction and bladder cancer are very frequent diseases. The same cannot be said about the association between these two pathologies [8].

We report a unique case of a patient admitted to the emergency room with a bowel obstruction caused by an aggressive urothelial carcinoma associated with an ileocolic fistula.

## Case presentation

An 80-year-old male, with an Eastern Cooperative Oncology Group (ECOG) performance status of 2, had been to the emergency room with the main complaint of anterior chest pain. The patient was a 20-pack-year smoker and had a history of type II diabetes, hypertension, dyslipidemia, and diabetic nephropathy with chronic kidney disease.

**Figure 1:**
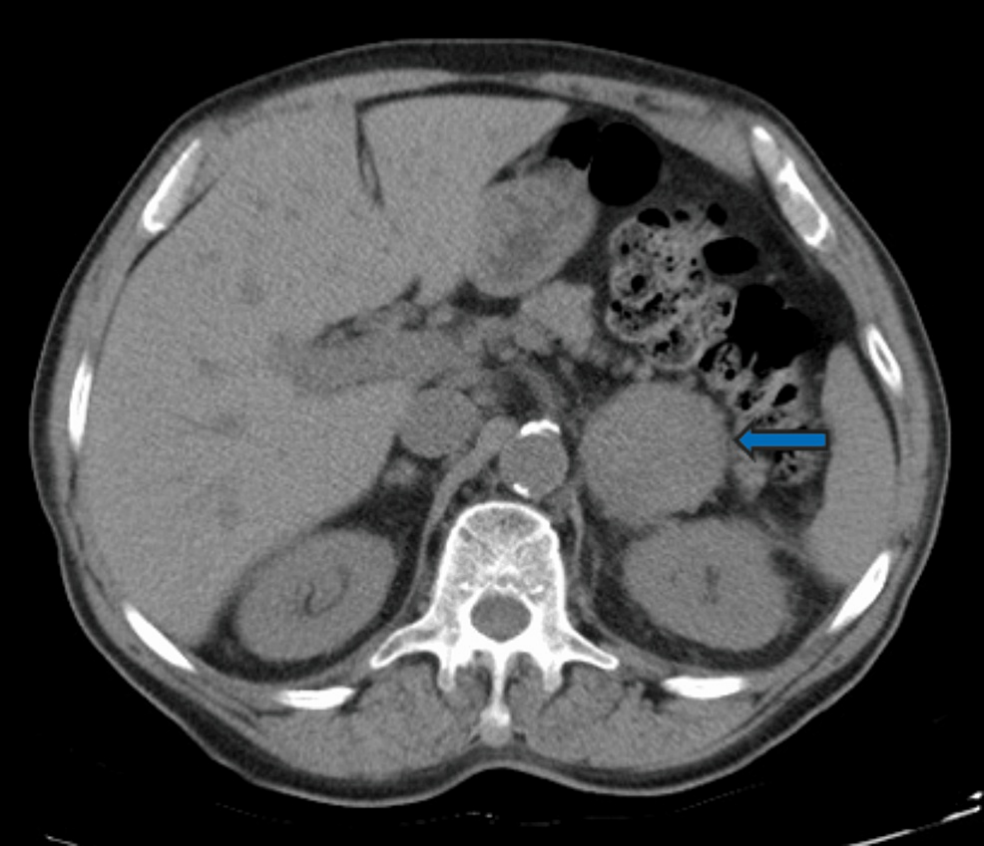
Adrenal lesion Blue arrow: Lesion of the left adrenal gland

During the workup, he was diagnosed with anemia (Hb 5 g/L) and underwent a thoracic computed tomography. The relevant showings included a 10 mm pulmonary nodule of the inferior lobe of the left lung and a 48 mm left adrenal lesion with a calcic center, suspected of metastatic nature (Figure 1).

Three units of packed red blood cells were administered with a good response, and the patient was discharged after excluding myocardial infarction. He was referred to the internal medicine consultation, where the location of the cancer of unknown origin was investigated.

**Figure 2:**
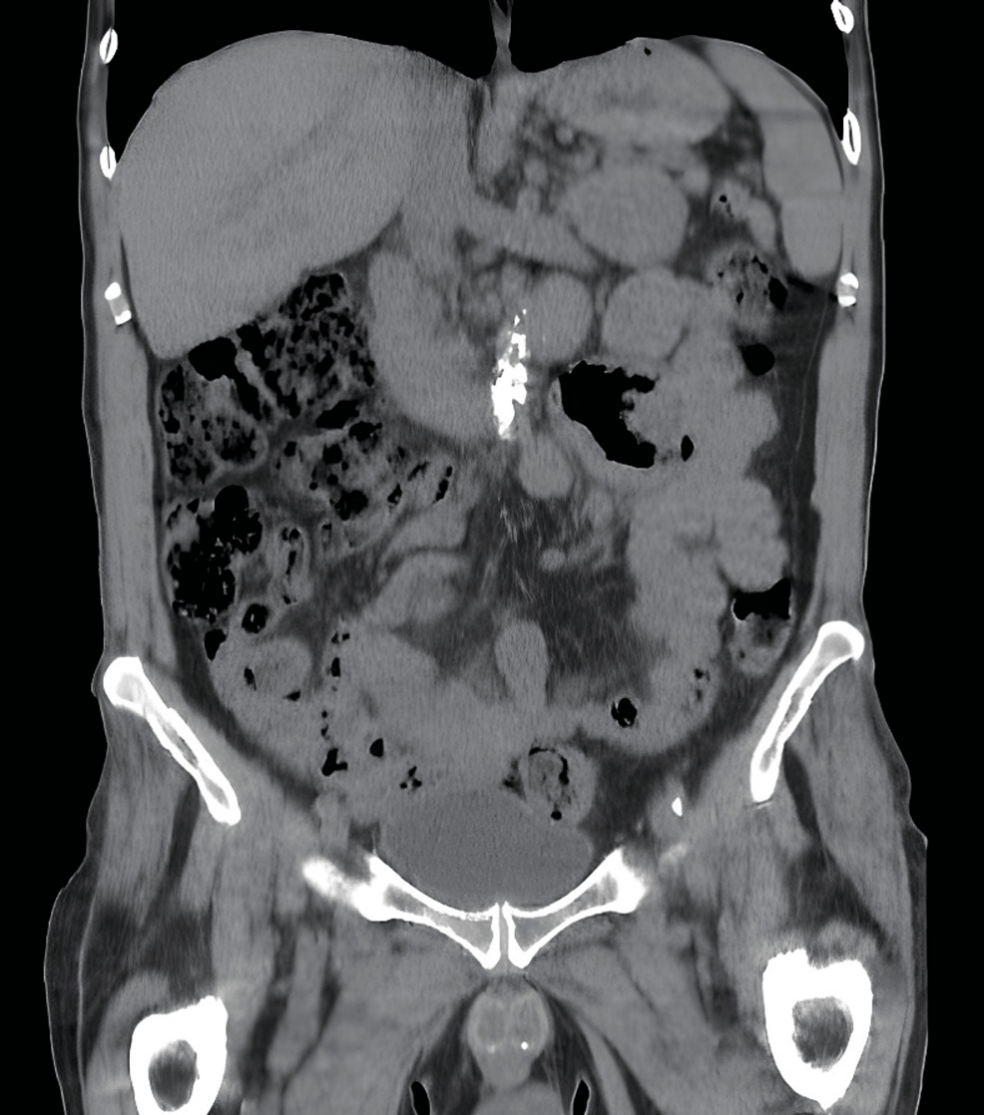
Abdominal CT scan

At first, an abdominopelvic CT scan was performed to complement the previous scan. This exam was performed without enhanced endovascular contrast due to chronic kidney disease (CKD) and did not reveal any new findings (Figure 2).

**Figure 3:**
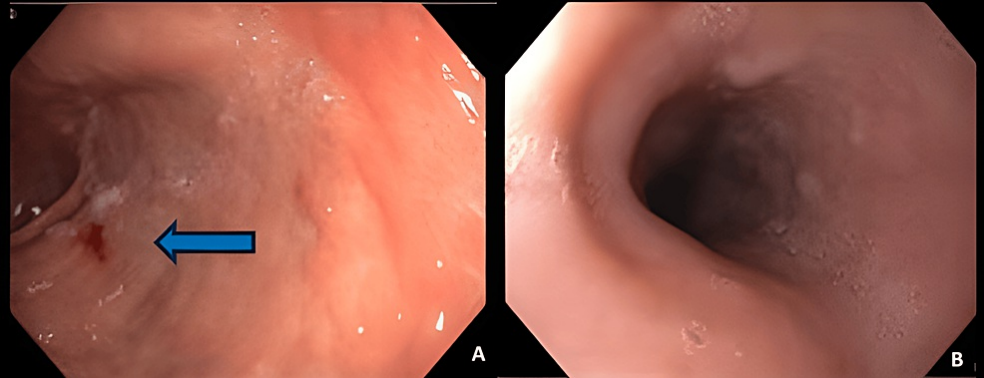
Upper endoscopy A: Left image showing signs of chronic gastritis (blue arrow); B: right image showing a normal gastric mucosa

To further investigate the anemia, the patient underwent an upper gastrointestinal endoscopy that was unremarkable, aside from the fact that it showed chronic gastritis of the antrum (Figures 3A, 3B).

**Figure 4:**
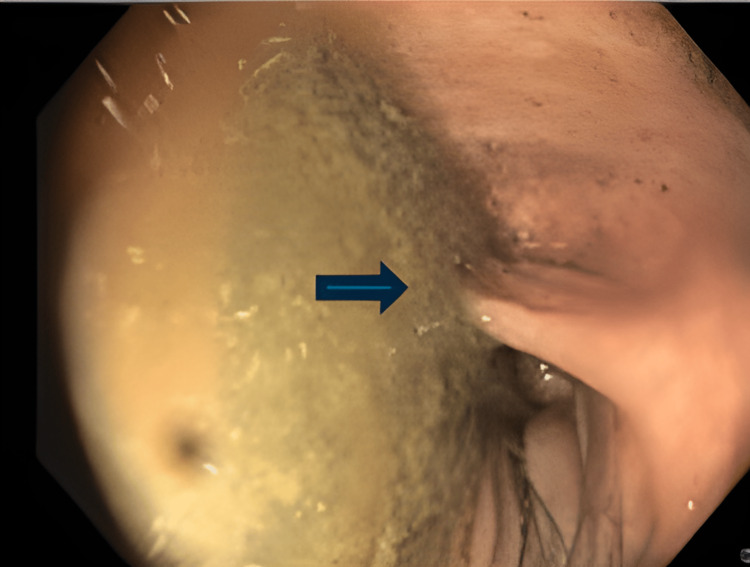
Colonoscopy findings Blue arrow: angulation of the sigmoid colon

He later underwent two colonoscopies. The first was not feasible without sedation due to the patient's lack of tolerability and the second colonoscopy detected a difficult-to-pass angulation of the sigmoid colon with no other abnormal aspects (Figure 4).

Magnetic resonance imaging of the adrenal glands was also performed to better clarify the adrenal lesion, which reinforced the hypothesis of a metastatic nature.

**Figure 5:**
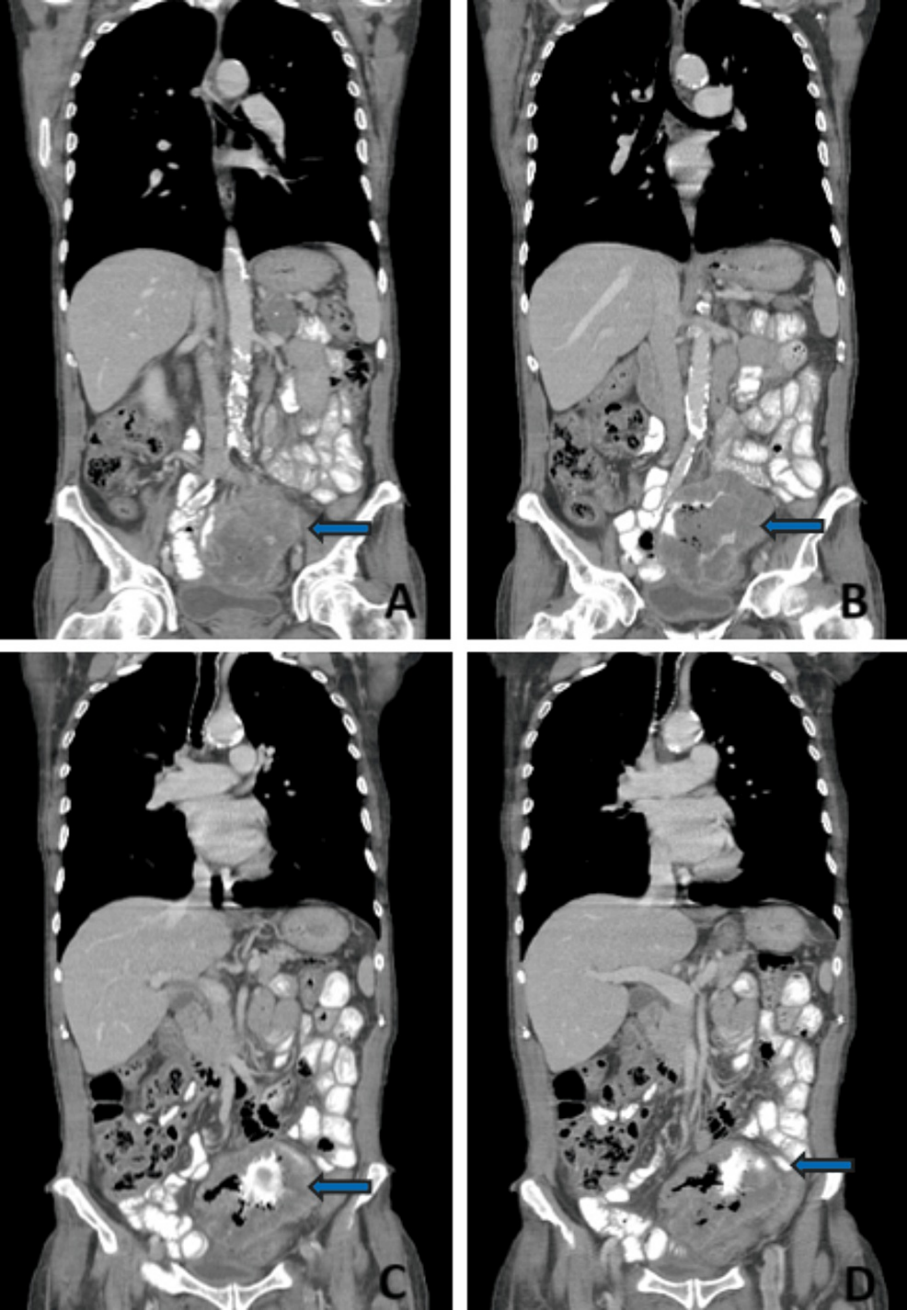
Thoracoabdominopelvic CT scan, coronal cuts A, B, C, and D: posterior to anterior coronal cuts; blue arrows: abdominal mass with an ileocolic fistula

**Figure 6:**
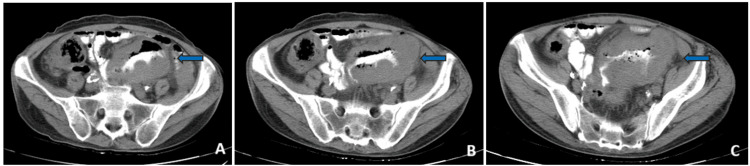
Thoracoabdominopelvic CT scan, transverse cuts A, B, and C: superior to inferior transverse cuts; Blue arrows: abdominal mass with an ileocolic fistula

Since no abnormal findings were reported, an endovascular and oral contrast-enhanced thoraco-abdominopelvic CT scan was performed. An abdominal mass involving the ileum and sigmoid colon in contact with the urinary bladder was observed and there was endoluminal contrast communicating between the ileum and the sigmoid colon, suggesting a fistula formation (Figures 5A-5D and Figures 6A-6C).

This patient was referred to the general surgery consultation and scheduled to be observed two days after referral. On the next day, he was admitted to the ER with diffuse abdominal pain and an absence of gastrointestinal transit in the prior two days.

A physical examination revealed a distended abdomen with general tenderness and a tympanic temperature of 38.5 ºC. The digital rectal examination was normal, with an empty rectal ampulla and without the presence of feces on the examining finger. No other significant aspects were found and a nasogastric tube was inserted with immediate drainage of 400 cc of stasis content.

**Figure 7:**
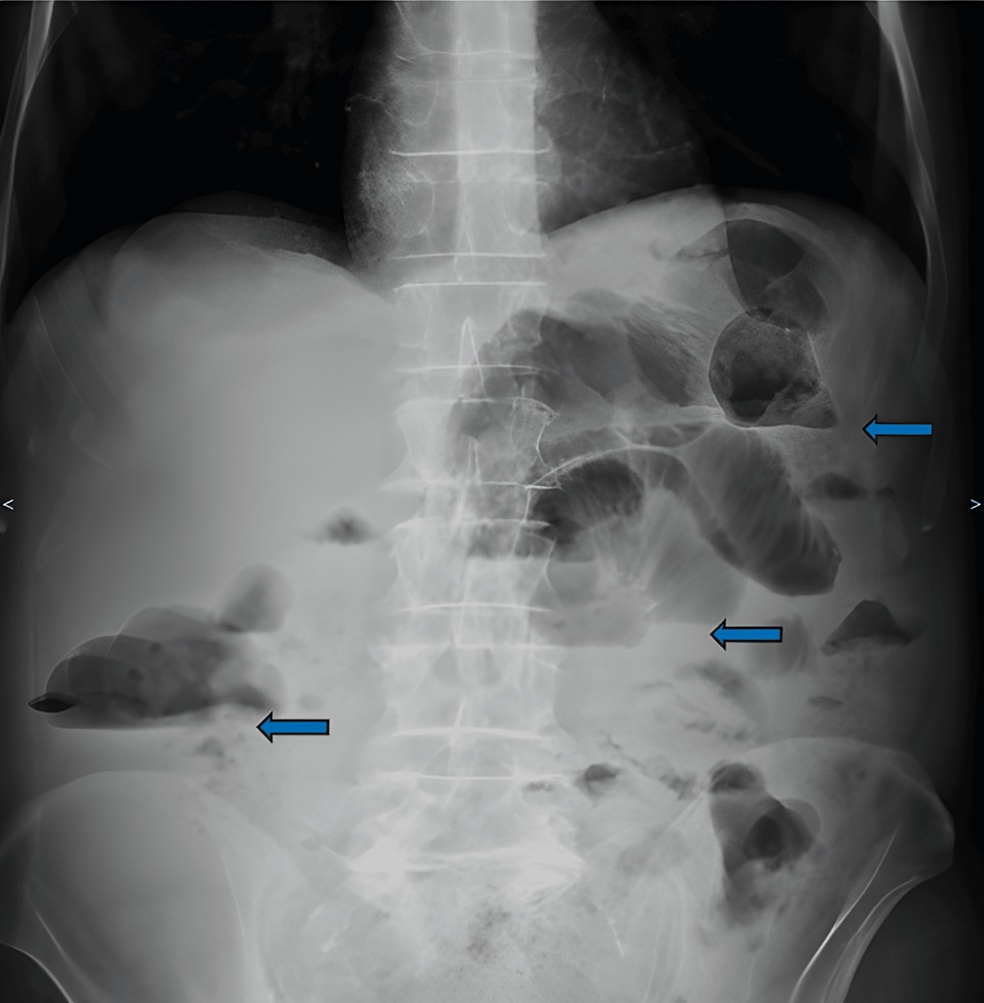
Abdominal X-ray Blue arrows: air-fluid levels

Laboratory values described an elevated C-reactive protein (CRP) level of 18.7 mg/dL with no other abnormalities, and the abdominal X-ray revealed multiple air-fluid levels (Figure 7).

**Figure 8:**
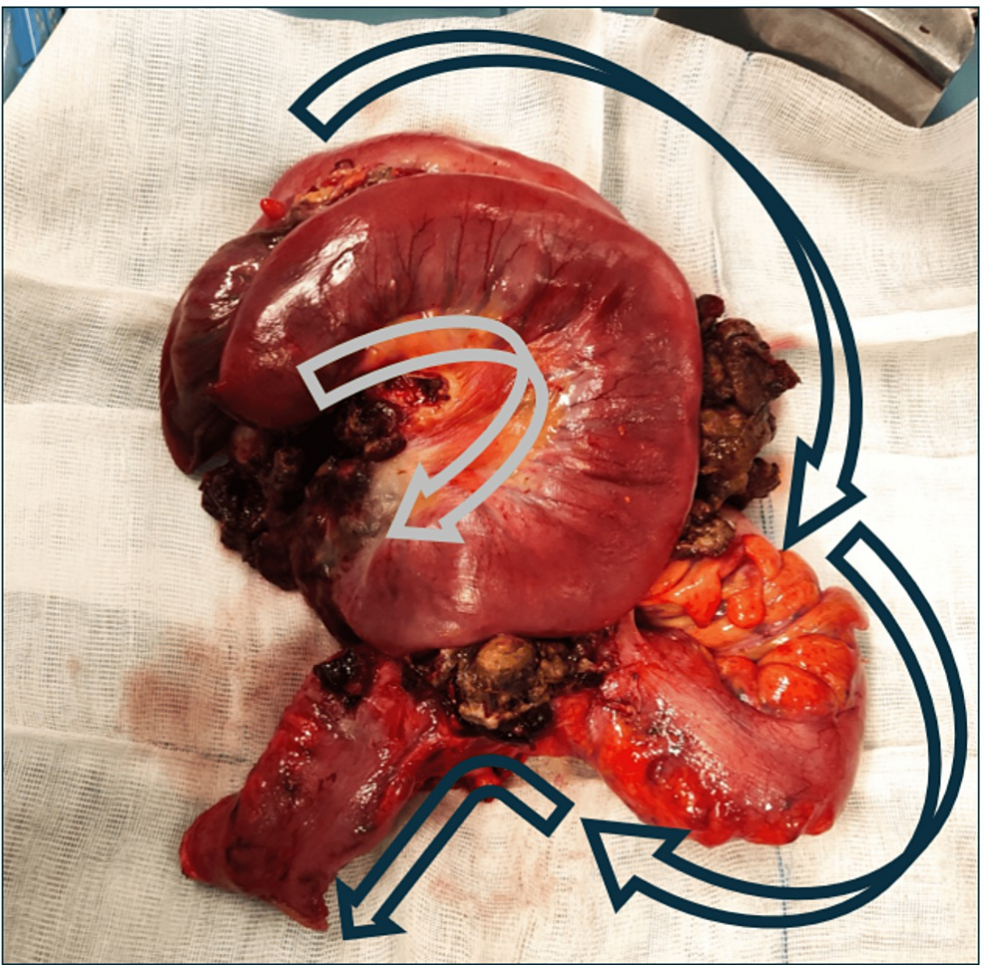
En-bloc resection specimen Gray arrow: small bowel and peristaltic direction; Blue arrow: sigmoid colon and peristaltic direction

The patient underwent an urgent laparotomy. Intraoperatively, an inspection of the peritoneal cavity confirmed a large tumorous mass suspected of gastrointestinal etiology. The mass infiltrated the ileum and sigmoid colon and seemed to be contacting the bladder wall. An en-bloc resection of the lesion that included the part of the descendent, the sigmoid colon, and 35 cm of the ileum was performed (Figure 8). An enteroenteric anastomosis and a terminal colostomy were performed. An R0 excison was not possible and fragments of the lesion were excised from the bladder wall for separate analysis. A large lymphadenopathy present in the mesenteric artery root was left in situ. There were no other findings.

The postoperative course was complicated with a postoperative ileus and a left pleural effusion with the need for thoracic drainage. There was a deterioration of the performance status with increased cachexia during the hospital stay and the patient was discharged on the twenty-ninth postoperative day with an otherwise uneventful stay.

Histopathological examination of the resected specimen described a high-grade, undifferentiated urothelial carcinoma that originated in the bladder and invaded the ileum and sigmoid colon. There were extensive tumoral deposits in the mesenteric fat and 10 mesenteric lymph nodes free of malignant disease. Immunohistochemical markers included: CK8/18+; CK7+; GATA3+; CD10+; KI67+ (90%); CK20-; CDX2-; CD45-; CD20-; CD3-; CD5-; CD23-; CD15-; CD30-; BCL2-; BCL6-; CIC-D1-; VIM-; CD56-.

The urothelial carcinoma of the bladder was staged pT4bNxcM+ and in the multidisciplinary group consultation, the patient was deemed unfit for more surgical or systemic treatments and is now in palliative care.

## Discussion

Worldwide, BC is the seventh most common cancer. It mainly affects Caucasians, and disease incidence increases with age and smoking habits [8-14]. As stated, although bowel obstruction and bladder cancer are very frequent diseases, these two entities are unlikely to be associated.

Urothelial carcinoma of the bladder does not usually spread by direct invasion and even more rarely presents with bowel obstruction, thus making this case particularly unique. However, as highlighted in this article, more advanced diseases may present in an unusual fashion [15].

This type of cancer is a malignancy of older adults aged over 60 that commonly presents with hematuria and infection or obstruction of the urinary tract [16]. About 85% of patients with urothelial carcinomas present with gross or microscopic hematuria at the diagnosis [17]. Other less frequent symptoms include disuria, poliaquiuria, constitutional symptoms, and weight loss [16]. All of these are symptoms that our patient never had, which probably added to the difficult diagnosis and the not-very-evident development of the tumor. 

The stage is the most important independent prognostic variable for the progression and overall survival of invasive bladder cancer [18]. The most important prognostic determinant that is derived from staging is whether the tumor is organ-confined (≤T2) or non-organ-confined (≥T3). The TNM system differentiates tumors extending into adjacent organs (T4) from those extending into perivesical fat (T3) [18]. Since this tumor invaded the ileum and colon, it is classified as a T4b, which proves to be detrimental to the patient’s outcome with a 5-year overall survival of less than 15% [16].

In the 2017 TNM (tumor, node, metastasis) staging system, a single lymph node metastasis in the true pelvis is considered N1 disease, multiple nodes in the true pelvis are classified as N2 disease, and nodal involvement of the common iliac nodes is classified as a secondary lymphatic drainage area (N3) rather than metastatic disease. Lymph node sampling should include the excision of an average of >12 lymph nodes. Despite not finding invasion in any of the 10 resected lymph nodes, it is not possible to provide an adequate N staging since a pelvic lymphadenectomy was not performed. A histological or cytological exam of the pulmonary nodules and adrenal gland was not achieved. Even though there is insufficient evidence, it is wise to assume an M+ staging in this case.

Besides TNM staging, the prognosis of UC depends on multiple factors. Variants with poor prognosis include urothelial carcinoma with rhabdoid features, urothelial micro-papillary carcinoma, plasmacytoid carcinoma, sarcomatoid carcinoma, small cell carcinoma, and, as outlined here, undifferentiated carcinoma [16]. Other poor prognostic factors, including lymphovascular invasion and large tumor size [16], are also presented in this patient.

Intestinal obstruction due to cancer represents a challenging matter in terms of diagnosis, life-saving strategies, obstruction resolution, and oncologic principles [14] as depicted by this case. An ileocolic fistula is a clinical presentation that might occur in patients with cancer or an inflammatory disease such as colorectal cancer or diverticulitis [18-19]. Even though this was not the focus of this article, it is important to note that, to our knowledge, to date, there has been no report of an ileocolic fistula caused by the invasion of bladder cancer.

## Conclusions

This article highlights a case of an aggressive urothelial carcinoma with an initial presentation of bowel obstruction and an ileocolic fistula. A thorough investigation of the patient showed very few results, suggesting this was an aggressive malignancy with rapid growth. Although the treatment options could include resection of the tumor and cystectomy in an earlier to moderately advanced disease, this patient intervention was intended as life-saving, and the later-achieved diagnosis was not suspected at the time. We also add that the advanced stage of the disease and the poor performance status of the patient could not allow for more complex surgery, as proved by the multidisciplinary group decision.

When dealing with bowel obstruction it is important to act accordingly, considering the most probable etiology. The presence of an invasive urothelial carcinoma presenting with bowel obstruction represents an unexpected diagnosis and, although rare, the surgeon must be aware of this possibility. This case should also serve as a reminder that a broad differential diagnosis should be considered when investigating an abdominal tumor.
